# Evaluation of light-emitting diodes as attractant for sandflies (Diptera: Psychodidae: Phlebotominae) in northeastern Brazil

**DOI:** 10.1590/0074-02760150132

**Published:** 2015-09

**Authors:** Francinaldo Soares Silva, Jefferson Mesquita Brito, Benedita Maria Costa, Shelre Emile Pereira Duarte Lobo

**Affiliations:** 1 Universidade Federal do Maranhão, Centro de Ciências Agrárias e Ambientais, Laboratório de Entomologia Médica, Chapadinha, MA, Brasil; 2 Universidade Federal do Maranhão, Programa de Pós-Graduação, Rede de Biodiversidade e Biotecnologia da Amazônia, São Luís, MA, Brasil

**Keywords:** light trapping, vector control, sandfly surveillance

## Abstract

Hoover Pugedo light traps were modified for use with green and blue-light-emitting diodes to trap phlebotomine sandflies in northeastern Brazil. A total of 2,267 specimens belonging to eight genera and 15 species were sampled. The predominant species were *Nyssomyia whitmani*(34.41%) and* Micropygomyia echinatopharynx*(17.25%).The green LED trap prevailed over the blue and control lights; however, no statistically significant difference could be detected among the three light sources. Even without statistical significance, we suggest using LEDs as an attractant for the capture of sandflies because of several advantages over the conventional method with incandescent lamps.

Phlebotomine sandflies are small dipteran insects that are distributed worldwide. The bite of the blood-feeding females is responsible for the transmission of several pathogens among vertebrate hosts, e.g., protozoan *Leishmania* species, the aetiological agents of the leishmaniasis disease (Ready 2013). Approximately 56 species of New World (America) phlebotomine sandflies are involved in the transmission of *Leishmania* species (Maroli et al. 2013, Brazil et al. 2015). In northeastern Brazil, *Nyssomyia whitmani*(Antunes & Coutinho, 1939) and *Lutzomyia longipalpis *(Lutz & Neiva, 1912) are the main vectors of *Leishmania *and are responsible for cutaneous and visceral leishmaniasis, respectively (Silva et al. 2010).

Phlebotomine vector control is a very important component of many antileishmaniasis programs (Alexander 2000). Sandflies are diurnally resting, crepuscular or nocturnally active species. Adults are attracted to artificial light sources, which has led to the widespread use of visible light-based traps for monitoring and mass trapping sandfly species. The Centers for Disease Control (CDC) miniature light traps have long been recognised as the most effective and widely used battery-operated traps for sampling medically important insects, including phlebotomine sandflies, in surveillance studies (Faiman et al. 2009). CDC light trap methods have become more effective over time, such as with the use of attractant-baited and light-emitting diode (LED) light traps (Mann et al. 2009, Cohnstaedt et al. 2012, Müller et al. 2014). LEDs with various wavelengths as excitation sources for attracting insects have recently been employed using modified light traps (Bishop et al. 2004, Hoel et al. 2007, Jenkins & Young 2010). There are some advantages, such as cheaper cost, difficulty to shatter, great durability and rare need of replacement, to the general use of LEDs as substitutes for the usual incandescent light sources. Furthermore, incandescent lamps emit light in the infrared range of the spectrum and most (95%) of this energy is emitted as heat (Cohnstaedt et al. 2008), which is invisible to most insects (Briscoe & Chittka 2001).

The use of LEDs to attract insects of medical importance has recently been reported, e.g., for the capture of phlebotomine sandflies and *Culicoides *biting midges, showing a preferential attraction of species to specific wavelengths of light (Bishop et al. 2004, Hoel et al. 2007, Silva et al. 2015). To the best of our knowledge, no comparative research has been carried out in Brazil on the effectiveness of LEDs as substitutes for incandescent lamps in the sandfly collection. This study was therefore conducted to determine the response of some Brazilian sandfly species to LEDs compared to incandescent lamps as an effective low-cost strategy for improving insect trapping efficiency.

The samples were collected from two forested areas in northeastern state of Maranhão (MA), Brazil. Adult sandflies were trapped in one area between November-December 2013 (5 nights) (3º45’46’’S 43°12’44’’W - near the community of Riacho Seco) and in another area between August 2014-January 2015 (14 nights) (3º50’40’’S 43º15’13’’W - near the community of Buritizinho). The two areas were 10 Km from each other. In the region, the climate is tropical and semihumid, with a well-defined wet season between January-June, followed by a drought period until the next rains. The annual average rainfall is 1,500-1,800 mm and the average temperature ranges from 28-30ºC.

Sandflies were sampled from 06:00 pm-06:00 am with three Hoover Pugedo light traps (Pugedo et al. 2005), two of which were modified. In the present study, the only modification was the replacement of the incandescent light bulbs by LEDs (1 LED for each trap). The modified traps held a 5-mm high-intensity LED bulb (Molesmell Technology Co, Ltd, China), one in the green (520 nm, 15,000 mCD, 20-30º, 20 mA, 72 mW - MLL5G30-1416) and the other in the blue (470 nm, 6,000 mCD, 20-30º, 20 mA, 72 mW - MLL5B30-0608) region of the visible spectrum. These colours were chosen from a set of six that were tested in a previous pilot study (data not shown) as the most attractive light sources for sandflies. An incandescent bulb (150 mA, 3 V) was used as a control. Light traps were set 1.5 m above the ground and were spaced 50 m from each other. The trap placement was randomised before each collecting day.

Sandflies were processed and identified to the species level according to Galati (2003). The specimens of*Micropygomyia echinatopharynx* were identified according to Andrade Filho et al. (2004). The collected specimens were deposited in the entomological collection of the Federal University of Maranhão, MA. Data were analysed using an ANOVA followed by pair-wise comparisons using the Student’s *t* test. Data were log transformed prior to analyses to achieve a more symmetric distribution. The differences between the averages are statistically significant when p < 0.05. The statistical analyses were performed using GraphPad Prism software (GraphPad Software Inc, USA).

A total of 2,267 specimens belonging to eight genera and 15 species were sampled using the three light traps (Table). The predominant species was *N. whitmani*, accounting for 34.41% of the collected specimens, followed by *M. echinatopharynx *(Andrade Filho, Galati, Andrade & Falcão, 2004) (17.25%), *Micropygomyia trinidadensis*(Newstead, 1922) (11.87%), *Lu. longipalpis* (8.60%),*Evandromyia termitophila *(Martins, Falcão & Silva, 1964) (8.25%), *Evandromyia evandroi* (Costa Lima & Antunes, 1936) (7.41%) and *Evandromyia lenti *(Mangabeira, 1938) (5.96%). The others accounted for 6.26%.

**TABLE:** Phlebotomine sandflies collected from light-emitting diode modified light traps in northeastern Brazil

Species	Green	M:F	Blue	M:F	Control	M:F	n (%)
*Nyssomyia whitmani*	327	1.6:1	274	1.4:1	179	1:1	780 (34.41)
*Micropygomyia echinatopharynx*	121	1:8	128	1:7.5	142	1:4.5	391 (17.25)
*Micropygomyia trinidadensis*	111	1.5:1	74	1.1:1	84	1.4:1	269 (11.87)
*Lutzomyia longipalpis*	55	1:1	55	1:1.3	85	1:1	195 (8.60)
*Evandromyia termitophila*	79	1.4:1	68	1.7:1	40	1:1	187 (8.25)
*Evandromyia evandroi*	63	1:1.9	69	1:4.1	36	1:2.2	168 (7.41)
*Evandromyia lenti*	53	1.6:1	54	2.7:1	28	4.6:1	135 (5.96)
Phlebotominae sp.	39	0:39	23	0:23	14	0:14	76 (3.35)
*Evandromyia saulensis*	9	1:3.5	13	0:13	5	1:1.5	27 (1.19)
*Sciopemyia sordellii*	9	2:1	15	1.1:1	2	0:2	26 (1.15)
*Bichromomyia flaviscutellata*	2	0:2	-	-	4	0:4	6 (0.26)
*Brumptomyia *sp.	1	0:1	1	0:1	2	0:2	4 (0.18)
*Psathyromyia *group*dreishbachi*	-	-	-	-	1	0:1	1 (0.04)
*Psathyromyia shannoni*	-	-	-	-	1	0:1	1 (0.04)
*Psathyromyia hermanlenti*	-	-	1	1:0	-	-	1 (0.04)

Total	869	-	775	-	623	-	2,267 (100)

control: incandescent lamp; M:F: male/female sex ratio.

The most frequent species shown here corroborates the finding of previous studies for northeastern MA (Silva et al. 2010, 2012). One remarkable exception is the presence of*M. echinatopharynx,* which was recently described from the state of Tocantins, Brazil (Andrade Filho et al. 2004).*M. echinatopharynx* was the second-most-common species in the samples and was found only in the Buritizinho.

The green light source represented 38.33% of the sandflies that were collected in the field, followed by blue (34.18%) and incandescent light (27.48%). Although the green LED was the most prevalent wavelength of light attracting sandflies to light traps, there was no statistically significant difference between the numbers of the three light sources that were used here (F_2 54_ = 0.5909, p = 0.5574). All of the species were LED biased, with the exception of *M. echinatopharynx* and*Lu. longipalpis*. In addition to these results, there was no significant difference between the most frequent species and the tested light sources.

The green-biased response was expected to occur because nocturnally active insects, such as sandflies, have trichromatic colour vision with photoreceptor sensitivities detecting the ultraviolet, blue and green wavebands (Briscoe & Chittka 2001, Mellor & Hamilton 2003). Previous studies examining the attraction of blood-sucking insects to light of various wavelengths have shown that individual responses to light sources may occur (Bishop et al. 2004, Silva et al. 2015). Hoel et al. (2007) found that *Phlebotomus papatasi* was strongly attracted to red light, followed by the control, blue and green lights and these results were defined by the authors as unexpected. It is possible that the intense brightness of the LEDs that were tested in our study accounted, at least in part, for the different results obtained with green and blue lights (1,500 and 650 mCD, respectively) compared to the control in the study by Hoel et al. (2007). Further studies are needed to better understand the role of brightness differences in the LEDs in attracting adult flying insects.

Our results showed that LED traps were as efficient as and even superior to incandescent lights for collecting sandflies. Thus, we suggest using LEDs as attractants for sandfly captures because this technology provides the above-mentioned advantages over the conventional method with incandescent lamps. Moreover, LEDs provide a much longer battery life, overcoming one of the constraints in conventional CDC light trapping. Further studies are needed to improve the use of LED light-trap catches in order to offer better alternatives for sandfly monitoring purposes.
